# Bioinformatic Analyses of Canonical Pathways of TSPOAP1 and its Roles in Human Diseases

**DOI:** 10.3389/fmolb.2021.667947

**Published:** 2021-06-15

**Authors:** Sharad Kumar Suthar, Mohammad Maqusood Alam, Jihye Lee, Jitender Monga, Alex Joseph, Sang-Yoon Lee

**Affiliations:** ^1^ Neuroscience Research Institute, Gachon University, Incheon, South Korea; ^2^ Manipal College of Pharmaceutical Sciences, Manipal University, Manipal, India; ^3^ Medicinal Chemistry, Institut Pasteur Korea, Seongnam, South Korea; ^4^ Department of Urology, Postgraduate Institute of Medical Education and Research, Chandigarh, India; ^5^ Department of Neuroscience, College of Medicine, Gachon University, Incheon, South Korea

**Keywords:** TSPO, TSPOAP1, IPA, canonical pathway analysis, mitochondrial dysfunction, neuroinflammation

## Abstract

TSPO-associated protein 1 (TSPOAP1) is a cytoplasmic protein and is closely associated with its mitochondrial transmembrane protein partner translocator protein (TSPO). To decipher the canonical signalling pathways of TSPOAP1, its role in human diseases and disorders, and relationship with TSPO; expression analyses of TSPOAP1- and TSPO-associated human genes were performed by Qiagen Ingenuity Pathway Analysis (IPA). In the expression analysis, necroptosis and sirtuin signalling pathways, mitochondrial dysfunction, and inflammasome were the top canonical pathways for both TSPOAP1 and TSPO, confirming the close relationship between these two proteins. A distribution analysis of common proteins in all the canonical pathways predicted for TSPOAP1 revealed that tumor necrosis factor receptor 1 (TNFR1), vascular cell adhesion molecule 1 (VCAM1), cyclic AMP response element-binding protein 1 (CREB1), T-cell receptor (TCR), nucleotide-binding oligomerization domain, leucine-rich repeat and pyrin domain containing 3 (NLRP3), DNA-dependent protein kinase (DNA-PK or PRKDC), and mitochondrial permeability transition pore (mPTP) were the major interaction partners of TSPOAP1, highlighting the role of TSPOAP1 in inflammation, particularly neuroinflammation. An analysis of the overlap between TSPO and TSPOAP1 *Homo sapiens* genes and top-ranked canonical pathways indicated that TSPO and TSPOAP1 interact *via* voltage-dependent anion-selective channels (VDAC1/2/3). A heat map analysis indicated that TSPOAP1 has critical roles in inflammatory, neuroinflammatory, psychiatric, and metabolic diseases and disorders, and cancer. Taken together, this information improves our understanding of the mechanism of action and biological functions of TSPOAP1 as well as its relationship with TSPO; furthermore, these results could provide new directions for in-depth functional studies of TSPOAP1 aimed at unmasking its detailed functions.

## Introduction

**FIGURE 1:**
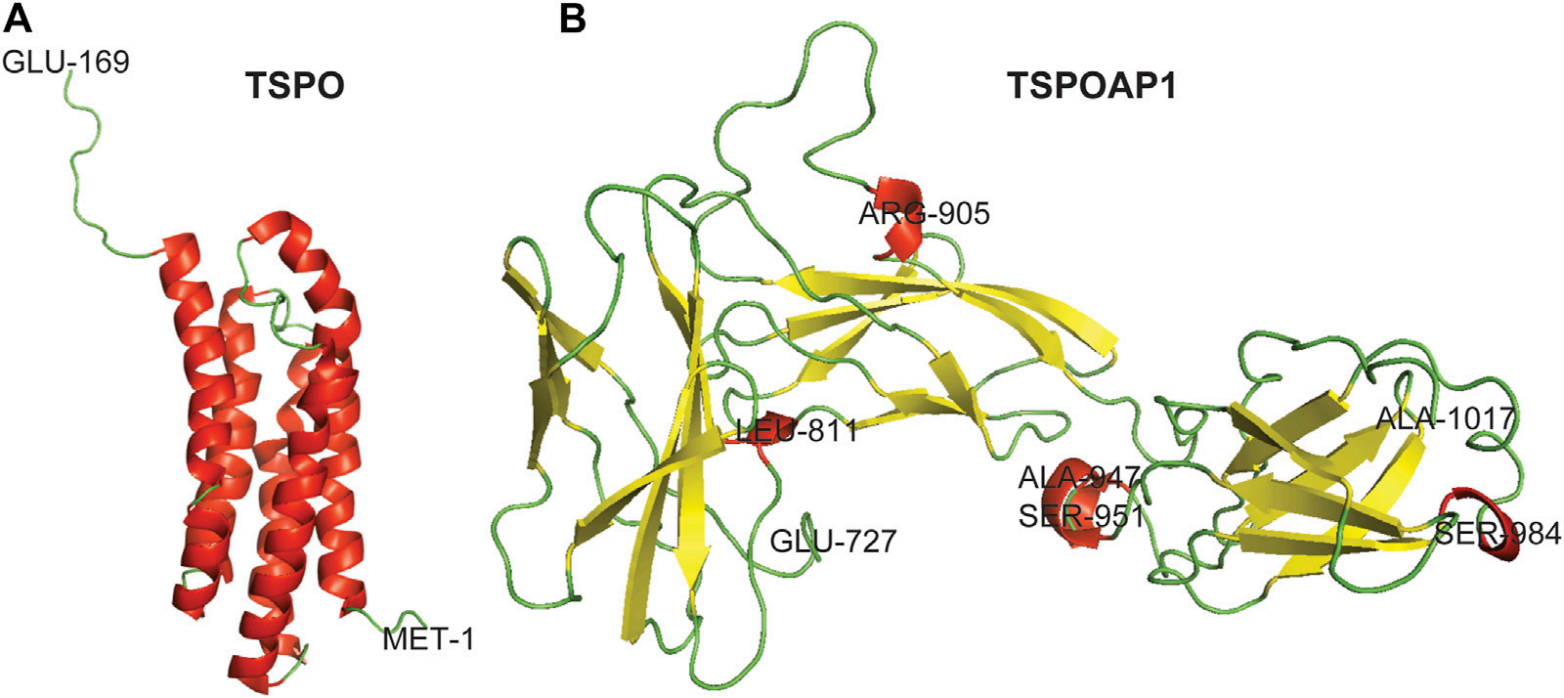
Three-dimensional crystal structures of TSPO and TSPOAP1. Helix–Red, Sheet–Yellow, and Loop–Green.

TSPO is an 18-kDa transmembrane protein of 169 residues predominantly localized in the outer mitochondrial membrane (Delavoie et al., 2003; Iacobazzi et al., 2017) (Figure 1A). It was first discovered in 1977 in a diazepam-binding site in the kidney and named peripheral-type benzodiazepine receptor (PBR) (Braestrup and Squires 1977). TSPO is a highly conserved ubiquitous protein with high abundance in steroid-producing tissues, including the glial cells, adrenal glands, testis, and ovary (Galiègue et al., 1999). The characteristic function of TSPO is cholesterol transport from the outer mitochondrial membrane to the inner membrane (Papadopoulos et al., 2007). Other functions of TSPO are the formation of mitochondrial permeability transition pore (mPTP) in conjunction with VDAC in the outer mitochondrial membrane, adenine nucleotide transporter (ANT) in the inner mitochondrial membrane, and cyclophilin D (Veenman et al., 2018; Dimitrova-Shumkovska et al., 2020). However, the role of TSPO, VDAC, and ANT in mPTP formation remains debated (Kokoszka et al., 2004; Baines et al., 2007; Šileikytė et al., 2014). On activation, TSPO generates reactive oxygen species that opens mPTP and release cytochrome C into the cytosol through the Bax-Bak channel, which leads to cell death (Garrido et al., 2006; Veenman et al., 2010; Veenman et al., 2018; Zeineh et al., 2019; Dimitrova-Shumkovska et al., 2020). The increased level of TSPO has been observed in neurodegenerative diseases and disorders (Rupprecht et al., 2010; Alam et al., 2017), brain injury (Papadopoulos and Lecanu 2009), and cancer (Decaudin et al., 2002; Li et al., 2007), while the dysregulation of TSPO has been detected in obesity (Kim et al., 2020) and diabetes (Giatti et al., 2009; Ilkan and Akar 2018).

TSPOAP1, also known as PBR-associated protein 1 (PRAX-1), is a cytoplasmic protein of 1857 residues (Figure 1B), was discovered in 1999 (Galiègue et al., 1999). As is evident from the name, TSPOAP1 specifically interacts with TSPO (Galiègue et al., 1999), and their functions and clinical applications overlap with each other. The modulation of TSPOAP1 activity has been implicated in neurodegenerative diseases and disorders (Blasi 2000; Jun et al., 2017; Witoelar et al., 2018), cancer (Galiègue et al., 1999), and obesity (Yim et al., 2020). The biological functions of TSPOAP1 are thought to be similar to those of its counterpart TSPO. Although it has been two decades since its discovery, very little is known about the functions and interaction partners of TSPOAP1 and its interplay with TSPO. The lack of canonical signalling information for TSPOAP1 limits molecular biology studies at various levels and the development of ligands as biological tools and therapeutics.

In this study, we deciphered the canonical signalling pathways of TSPOAP1, its protein interaction partners, and its roles in human diseases and disorders. We also analyzed the relationship between TSPOAP1 and TSPO and their common and unique interacting proteins or genes, providing insight into their overlapping and distinct biological functions. As a whole, this work is useful for researchers focused on the detailed molecular mechanisms and biological functions of TSPOAP1 and provides a basis for laboratory experiments aimed at unravelling the complex relationship between TSPOAP1 and TSPO.

## Materials and Methods

### TSPOAP1 and TSPO Datasets

TSPOAP1 and TSPO genes available up to September 7, 2020 were searched in the National Center for Biotechnology (NCBI) database (NCBI, 2020) with the search terms “TSPO associated protein one” and “TSPO,” respectively, (Supplementary Tables S1, S2). The results were limited to the taxon *Homo sapiens* and were downloaded as text files for subsequent analyses.

### Building and Analysis of TSPOAP1 and TSPO Interaction Networks

The core analyses (interaction networks or interaction pathways) of TSPOAP1 and TSPO were built and analyzed using Ingenuity Pathway Analysis (IPA), Qiagen (IPA, 2020a). The gene file of the respective TSPOAP1 or TSPO protein obtained from the NCBI database was uploaded to the IPA server for the “core analysis” in the flexible format. The gene identification (GI) number was defined and the “expression analysis” as a type was chosen for the new core analysis. The population of genes considered for *p*-value calculations was restricted to the Ingenuity Knowledge Base only (defining the reference set), while opting for both direct and indirect relationships among genes affecting interaction networks and upstream regulators. For pre-analysis filtering of networks, for interaction networks; genes, including endogenous chemicals, 35 molecules per network and 25 networks per analysis were selected. For casual networks that relate to master regulators for relationships to diseases, functions, genes, or chemicals selection, were also ticked along with the option “score using casual paths only.” The node types (molecule types filter) in the IPA represent different canonical pathways, proteins, genes, endogenous and exogenous chemicals, diseases, drugs, and their biological functions and effects, which allows us to build pathways based upon the types we select. For core analyses, all node types available on the IPA server were selected. The data source in IPA allows us to select the particular data sets for our analysis. For the TSPOAP1 and TSPO core analyses, all data sources available on the IPA server were selected. Among other parameters for pathway building, only experimentally observed confidence was selected (filter for the selection of microRNAs involved in interaction networks) and species selection was restricted to humans only (filter for the selection of molecules and relationship to specific species). All types of tissues and cell lines as well as all kinds of mutations, such as functional, inherited, translation, zygotic, wild-type, and other unclassified mutations, were chosen to build the interaction networks (IPA, 2020b). The results were ranked based on Fisher’s exact test, where smaller *p*-values indicate a lower likelihood of a random association between the data set and predictions. GraphPad Prism 6 was used at various stages of the analyses to plot the results.

### Preparation of 3-Dimensional Crystal Structures of TSPO and TSPOAP1

The 3-D crystal structure of TSPO was downloaded from RCSB Protein Data Bank (PDB ID: 2MGY). The 3-D crystal structure of TSPOAP1 was prepared by homology modeling using the Swiss-Model server (Swiss-Model 2020). For this, the FASTA sequence of TSPOAP1 (UniProtKB ID: O95153, UniProt, 2020) isoform 1 (identifier: O95153-1) was submitted to the Swiss-Model server. The obtained models were ranked based on various parameters and the model with the highest sequence similarity was used for the presentation of the 3-D crystal structure of TSPOAP1. PyMOL was used for the visualization of the 3-D crystal structures of TSPO and TSPOAP1.

## Results

### Identification of Canonical Pathways Associated With TSPOAP1 and TSPO

**FIGURE 2:**
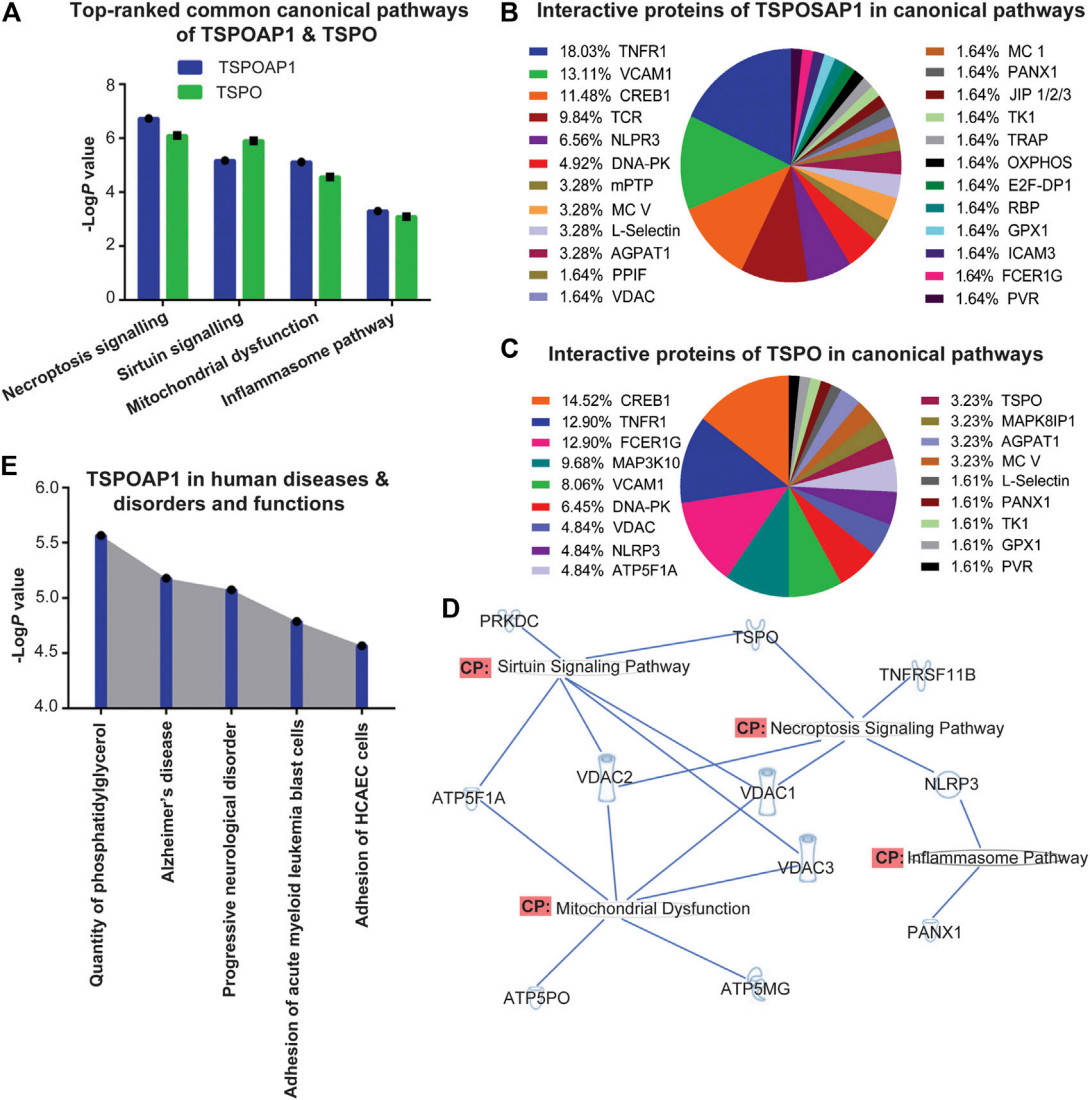
**(A)** Top-ranked common canonical pathways of TSPOAP1 and TSPO. **(B)** Interaction partners of TSPOAP1 in the canonical pathways. **(C**) Interaction partners of TSPO in the canonical pathways. **(D)** Overlapping of top-ranked canonical pathways of TSPOAP1, highlighting the common enzymes, proteins, and genes in these signalling pathways. **(E)** Top-five human diseases and disorders and functions associated with TSPOAP1. Symbols in Figure 2D: TSPO and TNFRSF11B; transmembrane receptor, VDAC 1/2/3; ion channel, NLRP3; complex/group/other, ATP5PO, ATP5F1A, and PANX1; transporter, ATP5MG; enzyme, PRKDC; kinase.

TSPOAP1 is closely associated with TSPO (Galiègue et al., 1999). Despite extensive studies of the mechanism of action and biological functions of TSPO (Lee et al., 2020), very little is known about the roles and functions of TSPOAP1 in human diseases and disorders. Accordingly, to characterize the functions of TSPOAP1 and its interactions with TSPO, we performed canonical pathway analyses, including expression analyses of signalling molecules related to both TSPOAP1 and TSPO using IPA. The canonical pathway analysis of *Homo sapiens* genes associated with TSPOAP1 (Supplementary Table S1) and TSPO (Supplementary Table S2) identified the necroptosis, sirtuin signalling, mitochondrial dysfunction, and inflammasome pathways as the major signalling pathways (Figure 2A and Supplementary Figures S1–S8). All the canonical pathways predicted by IPA for TSPOAP1 and TSPO are summarized in Table 1 and Supplementary Table S4, respectively, and in Supplementary Figure S1.

**TABLE 1:** Canonical pathways of TSPOAP1 predicted by Ingenuity Pathway Analysis, highlighting the interacting protein or gene partners in the signalling pathways.

Rank	Canonical pathway	-log(*p*-value)	*p*-value	Interacting pathway proteins/genes
1	Necroptosis signalling pathway	6.728	1.87E^−07^	- TNFR1
				- NLRP3 inflammasome or Caspase-1 inflammasome (Nalp3, Asc, Csp1)
				- PPIF and Mpt pore (mPTP)
2	Sirtuin signalling pathway	5.171	6.74E^−06^	- Mpt pore (mPTP)
				- OXPHOS – Mitochondrial complex 1 (NDUFA9, SDHA, ATP5*β*) (MC 1)
				- DNA-PK (PRKDC)
3	Mitochondrial dysfunction	5.115	7.67E^−06^	- VDAC
				- Mitochondrial complex V or mitochondrial ATP synthase ((MC V)
4	Inflammasome pathway	3.295	5.07E^−04^	- NLRP3 inflammasome or Caspase-1 inflammasome (Nalp3, Asc, Csp1)
				- PANX1
5	Granulocyte adhesion and diapedesis	2.582	2.62E^−03^	- TNFR1
				- VCAM1
				- L-Selectin
6	Dendritic cell maturation	2.523	3.00E^−03^	- TNFR1
				- TCR
				- CREB1
7	T Helper cell differentiation	2.225	5.95E^−03^	- TNFR1
				- TCR
8	SAPK/JNK signalling	1.907	1.24E^−02^	- TCR
				- JIP 1/2/3
9	Neuroinflammation signalling pathway	1.907	1.24E^−02^	- NLRP3 inflammasome or Caspase-1 inflammasome (Nalp3, Asc, Csp1)
				- CREB1
				- VCAM1
10	Role of macrophages, fibroblasts, and endothelial cells in rheumatoid arthritis	1.886	1.36E^−02^	- TNFR1
				- TNFR SF11B or OPG
				- CREB1
				- VCAM1
11	Oxidative phosphorylation	1.883	1.31E^−02^	- Mitochondrial complex V or mitochondrial ATP synthase (MC V)
12	Salvage pathways of pyrimidine deoxyribonucleotides	1.876	1.33E^−02^	- Thymidine kinase 1 (TK1)
13	Type 1 Diabetes Mellitus signalling	1.86	1.38E^−02^	- TNFR1
				- TCR
14	Estrogen receptor signalling	1.788	1.63E^−02^	- CREB1
				- TRAP/Mediator complex
				- OXPHOS
15	White adipose tissue browning pathway	1.712	1.94E^−02^	- E2F-DP1
				- CREB1
16	Hepatic fibrosis signalling pathway	1.654	2.22E^−02^	- TNFR1
				- CREB
				- VCAM1
17	DNA double-strand break repair by non-homologous end joining	1.635	2.32E^−02^	- DNA-PK (PRKDC)
18	Role of pattern recognition receptors in recognition of bacteria and viruses	1.616	2.42E^−02^	- NLRP3
				- CREB1
19	Granzyme B signalling	1.577	2.65E^−02^	- DNA-PK (PRKDC)
20	HMGB1	1.547	2.84E^−02^	- TNFR1
				- VCAM1
21	CDP-diacylglycerol biosynthesis I	1.481	3.3E^−02^	- 1-Acylglycerol-3-phosphate-O-acyltransferase (AGPAT1)
22	Agranulocyte adhesion and diapedesis	1.456	3.5E^−02^	- VCAM1
				- L-Selectin
23	NF-kB signalling	1.456	3.5E^−02^	- TNFR1
				- TCR
24	Acute phase response signalling	1.447	3.57E^−02^	- TNFR1
				- RBP
25	Glutathione redox reactions I	1.441	3.62E^−02^	- Glutathione peroxidase 1 (GPX1)
26	Phosphatidylglycerol biosynthesis II (non-plastidic)	1.441	3.62E^−02^	- 1-Acylglycerol-3-phosphate-O-acyltransferase (AGPAT1)
27	Hepatic fibrosis/Hepatic stellate cell activation	1.429	3.72E^−02^	- TNFR1
				- VCAM1
28	Lipid antigen presentation by CD1	1.405	3.94E^−02^	- TCR
29	Natural killer cell signalling	1.391	4.06E^−02^	- FCER1G
				- PVR
30	Leukocyte extravasation signalling	1.384	4.13E^−02^	- VCAM1
				- ICAM3

**TNFR1**, Tumor necrosis factor receptor 1; **NLRP3**, Nucleotide-binding oligomerization domain, leucine-rich repeat and pyrin domain containing 3; **ASC**, Apoptosis-associated speck-like protein containing a CARD; **Csp1**, Caspase-1; **PPIF**, Peptidylprolyl isomerase F; **Mpt pore (mPTP)**, Mitochondrial permeability transition pore; **NDUFA9**, NADH-ubiquinone oxidoreductase subunit A9; **SDHA**, Succinate dehydrogenase complex flavoprotein subunit 9; **ATP5*β***, ATP synthase F1 subunit *β*; **DNA-PK (PRKDC)**, DNA-dependent protein kinase; **PANX1**, Pannexin 1; **TCR**, T-cell receptor; **CREB**, Cyclic AMP response element binding protein 1; **JIP 1/2/3**, JNK-interacting protein 1/2/3; **VCAM1**, Vascular cell adhesion molecule 1; **OPG**, Osteoprotegerin; **TRAP/Mediator complex**, Thyroid hormone receptor-associated protein (TRAP)/Mediator coactivator complex; **OXPHOS**, Oxidative phosphorylation; **E2F**, E2 factor family of transcription factors; **DP1**, transcription factor dimerization partner; **AGPAT1**, 1-Acylglycerol-3-phosphate-O-acyltransferase; **RBP**, Retinol-binding protein; **FCER1G**, Fc fragment of IgE receptor Ig; **PVR**, PVR cell adhesion molecule; **ICAM3**, Intercellular adhesion molecule 3

### Analysis of Protein Interaction Networks of TSPOAP1 and TSPO

After predicting canonical pathways, we analyzed the interacting proteins of TSPOAP1 and TSPO in those pathways. In all the predicted canonical pathways, TNFR1, VCAM1, CREB1, NLRP3, and DNA-PK were the major and common interacting partners of both TSPOAP1 and TSPO (Figures 2B,C). Other common interacting partners between TSPOAP1 and TSPO were mitochondrial ATP synthase (MC V), L-selectin, AGPAT1, and VDAC. These common interacting partners suggest overlapping functions and roles of TSPOAP1 and TSPO. Since the most prominent signalling pathways were shared by both TSPOAP1 and TSPO (Figure 2A), we focused on the differences in proteins that interact with TSPOAP1 and TSPO in the canonical pathways. Upon distribution analysis of interacting proteins in the pathways, we observed that TCR and m-PTP were significantly populated in TSPOAP1 signalling, whereas MAP3K10 and FCERIG more frequently appeared in TSPO signalling. Furthermore, VDAC1/2/3, responsible for transport between the mitochondria and cytosol (Hiller et al., 2010), were also more frequent in the TSPO pathways.

### Identification of Cross-Talk Mediator Between TSPOAP1 and TSPO

To establish the relationship between TSPOAP1 and TSPO, we evaluated the overlap between their prominent signalling pathways (Figure 2A and Supplementary Figures S4–S8) and *Homo sapiens* genes associated with TSPOAP1 and TSPO (Supplementary Tables S1–S3). Overlapping of signalling cascades and genes revealed VDAC1/2/3 as the key mediators of the relationship between TSPOAP1 and TSPO (Figure 2D).

### Prediction of Pathological Conditions and Functions Associated With TSPOAP1 and TSPO

A heat map analysis by IPA was used to predict the major diseases and functions associated with TSPOAP1 and TSPO. It predicted quantity of phosphatidylglycerol, Alzheimer’s disease, progressive neurological disorder, adhesion of acute myeloid leukemia blast cells, and adhesion of human coronary artery endothelial cells (HCAEC) (Figure 2E and Supplementary Figures S9, S10) as the major diseases and functions associated with TSPOAP1. The major diseases and disorders predicted for TSPO were inflammatory, neuroinflammatory and psychiatric diseases and disorders, cancer, and metabolic syndrome (Supplementary Figures S11, S12).

## Discussion

### IPA Predicts Canonical Pathways of TSPOAP1 and TSPO

Our IPA-based canonical pathway analysis identified several signalling pathways associated with TSPOAP1 and TSPO. Based on their significance potential, necroptosis, sirtuin signalling, mitochondrial dysfunction, and inflammasome pathways were identified as the major signalling pathways of TSPOAP1 and TSPO. Necroptosis is a type of cell death, which occurs with a regulated mechanism like apoptosis (Galluzzi and Kroemer 2008). Neurodegenerative diseases are characterized by chronic neuroinflammation and neuronal death (Heckmann et al., 2019). Inhibition of necroptosis mediators leads to reduced tissue inflammation (Galluzzi and Kroemer 2008). The mechanisms of neuronal degeneration are not well understood; however, an extensive interplay between various pathways has been indicated (Heckmann et al., 2019). In our study, we found that the induction of TNF-α promotes necroptosis via downstream TNFR and RIP molecules (Supplementary Figure S4). In addition, activation of FASL and TLR was also observed during necroptosis (Supplementary Figure S4). Accordingly, the identification of molecular determinants of necroptosis may have major therapeutic implications in various diseases. Our analysis also predicted that TSPOAP1 and TSPO strongly regulate sirtuin signalling (Supplementary Figures S5, S6), which may contribute to sirtuins mediated control and regulation of metabolism, oxidative stress, and DNA damage in inflammation and cancer (Houtkooper et al., 2012; Edatt et al., 2020). Mitochondrial dysfunctionality is known to play dynamic roles in oxidative stress, apoptosis, metabolism, and immune responses (Nicolson 2014), was also found to be associated with TSPOAP1 and TSPO. It arises due to dysfunction in their ATP production and electron transport machinery leading to decreased ATP synthesis and increased reactive oxygen species, which ultimately results in metabolic syndrome and oxidative damage to tissues (Nicolson 2014). Our pathway analysis shows that TSPOAP1 and TSPO may regulate several key genes of mitochondrial complexes (Supplementary Figure S7), which play indispensable roles in mitochondrial dysfunction. Furthermore, the regulation of inflammasomes was identified to be linked with TSPOAP1 and TSPO (Supplementary Figure S8). Inflammasomes are the multiprotein complex of the innate immune system (NLRP3, NLRC4, AIM2, and caspase-11), which initiate an inflammatory response against foreign bodies and infections (Guo et al., 2015) (Supplementary Figure S8). Combining these pieces of information from the major signalling pathways of TSPOAP1 and TSPO, we can state that both proteins seem majorly involved in inflammation cascades and orchestrate a pivotal role in inflammation, oxidative stress, aging, cancer, and metabolic syndrome.

### IPA Reveals Interaction Molecules of TSPOAP1 and TSPO

After predicting canonical pathways, we analyzed those pathways to discover the common as well as exclusive interacting molecules of TSPOAP1 and TSPO. Statistical analyses of interacting proteins distributed in the canonical pathways of TSPOAP1 and TSPO revealed TNFR1, VCAM1, CREB1, NLRP3, and DNA-PK as the common interacting partners of both proteins (Figures 2B,C). TSPOAP1 and TSPO mediate the inflammatory feedback through TNFR1 and downstream NF-κB, a transcription factor that promotes inflammation and carcinogenesis (Suthar and Sharma 2016). TSPOAP1 and TSPO also alter the activity of VCAM1, a transmembrane protein which facilitates the adhesion of leukocytes to endothelial cells (Kong et al., 2018; GeneCards, 2020). Furthermore, TSPOAP1 and TSPO play a central role in inflammation, apoptosis, and immune response by modulating the response of NLRP3 inflammasome. NLRP3 interacts with apoptosis-associated proteins, like ASC, and is induced by TLR ligands, such as lipopolysaccharide via NF-κB signalling; (Cocco et al., 2014; GeneCards, 2020). In addition, TSPOAP1 and TSPO mediate the activity of CREB1, a transcription factor, which promotes cell proliferation and differentiation via association with cAMP response element modulatory protein (Crem) and activating transcription factor 1 (Atf1) (Antony et al., 2016; Friedrich et al., 2020). DNA-PK is a serine/threonine protein kinase, which acts as a molecular sensor against DNA damage and repairs it (Medová et al., 2020). Abnormal expression and deregulation of DNA-PK lead to inflammatory disorders and cancer (Medová et al., 2020). TSPOAP1 and TSPOP alter the response of DNA-PK against DNA damage. Taken together, the interaction of both TSPOAP1 and TSPO with these proteins supports the overlapping functions of TSPOAP1 and TSPO, including shared roles in inflammation, oxidative stress, metabolic syndrome, and cancer.

Besides the identification of common interaction partners of TSPOAP1 and TSPO, we also identified proteins that exclusively or favorably interact with either TSPOAP1 or TSPO, which could enable us in making the distinction between their functions. Our findings revealed that the percent distribution of TCR and mPTP were significantly higher in TSPOAP1 canonical pathways (Figure 2B) than TSPO pathways (Figure 2C). TCR is a protein that identifies foreign antigens on major histocompatibility complex (MHC) and activates T cells through signal transduction mechanism (Hwang et al., 2020); which may explain the key role of TSPOAP1 in the immune response. In addition, our analysis found mPTP significantly expressed in the TSPOAP1 pathways (Figure 2B) compared to TSPO pathways (Figure 2C), which was surprising considering the well-established association between the TSPO and mPTP (Veenman et al., 2018; Dimitrova-Shumkovska et al., 2020). The mPTP is an event that occurs in response to injury and inflammation (Lemasters et al., 2009; Kwong and Molkentin 2015). The formation of mPTP induces mitochondrial cell death (Kwong and Molkentin 2015). The molecular structure of mPTP still remains a puzzle and far from settled (Kwong and Molkentin 2015). Historically, various studies suggested that mPTP is consisting of VDAC, ANT, and CypD (Kwong and Molkentin 2015). TSPO was also speculated to be a part of the mPTP core (McEnery et al., 1992). However, recent genetic studies of each mPTP constitute have provided contrasting results. Studies have shown that VDAC^−/−^ mitochondria are still able to form mPTP (Baines et al., 2007) while genetic ablation of ANT also does not stop mitochondria from forming the mPTP (Kokoszka et al., 2004). The deletion of TSPO in the mouse model still leads to mPTP formation (Šileikytė et al., 2014). Henceforth, the proteins which exactly comprise an mPTP structure remain an area of investigation. Our results revealed that it is TSPOAP1 instead of TSPO that predominantly interacts with mPTP; opens a new research paradigm on TSPOAP1’s direct or indirect connection in the mPTP formation. We believe that further investigations on the role of TSPOAP1 in mPTP formation will immensely contribute to solving the conundrum of mPTP structure. At the same time, these studies will unmask the physiological and pathological role of TSPOAP1.

In the case of TSPO’s exclusive interacting partners, we observed a high to the relatively high population of MAP3K10 and FCER1G in the canonical pathways compared to TSPOAP1 signalling cascades (Figures 2B,C). MAP3K10 is stimulated in response to reactive oxygen species and inflammatory conditions; plays a critical role in the inflammation of brain tissues via the downstream JNK signalling pathway (Upadhyay et al., 2019). FCER1G is a component of high-affinity immunoglobulin E, mediates allergic inflammatory signalling through downstream MAPK, phosphatidylinositol, and NF-κB signalling cascade (Cusabio 2021; UniProt 2021). Thus, the high population of MAP3K10 and FCER1G proteins in TSPO signalling suggests that TSPO mediates inflammatory response perhaps more extensively than TSPOAP1.

### IPA Predicts Proteins Facilitating Communication Between TSPOAP1 and TSPO

To discover the molecules facilitating communication between TSPOAP1 and TSPO, we overlapped TSPOAP1 and TSPO signalling pathways (Figure 2A and Supplementary Figures S4–S8) and their genes (Supplementary Tables S1–S3). Upon overlapping of pathways, VDAC1/2/3 emerged as the major communication bridge between TSPOAP1 and TSPO (Figure 2D), suggesting that two proteins interact with each other via chemical messengers facilitated by VDAC. VDAC1/2/3 or porin ion channels transport cargos between mitochondria and cytosol (Tsujimoto and Shimizu 2002; Hiller et al., 2010), alter mPTP activity, and play a dynamic role in apoptosis (Tsujimoto and Shimizu 2002; Lemasters and Holmuhamedov 2006), suggesting that TSPOAP1 and TSPO may influence or modulate these functions.

### IPA Predicts Diseases and Functions Linked to TSPOAP1 and TSPO

Finally, this study identified diseases and functions associated with TSPOAP1 and TSPO. Based on the heat map analysis of diseases and functions, TSPOAP1 can be explored as a potential drug target for phosphatidylglycerol-regulated functions, Alzheimer's disease, progressive neurological disorder, adhesion of acute myeloid leukemia blast cells, and adhesion of human coronary artery endothelial cells (HCAEC) (Figure 2E and Supplementary Figures S9–S10). Phosphatidylglycerol is a lipid that forms cardiolipin, a component of the inner mitochondrial membrane (Pizzuto and Pelegrin 2020). Cardiolipin maintains the electron transport chain and the activity of complex I, III, and IV and ATP synthase (Pizzuto and Pelegrin 2020). TSPOAP1 deregulates phosphatidylglycerol and thereby impairs the activity of cardiolipin in the brain, which leads to progressive neurological disorders and cancer. TSPOAP1 through adhesion of acute myeloid leukemia cells promotes resistance to chemotherapy, including cytarabine (Becker 2012). Therefore, TSPOAP1 can be explored as a potential drug target for drug-resistant acute myeloid leukemia. Based on its role in the adhesion of human coronary artery endothelial cells, TSPOAP1 may be a critical factor in various diseases, like atherosclerosis, and the development of TSPOAP1 ligands is a potential strategy for the treatment of coronary artery disease. Likewise, TSPO was predicted to be involved in neuroinflammatory, inflammatory, psychiatric, and metabolic diseases as well as cancer (Supplementary Figures S11, S12). The involvement of TSPO in these diseases was expected because TSPO generates reactive oxygen species that form mPTP - the enigmatic gatekeeper of apoptosis and necroptosis, decreases ATP synthesis, and eventually results in cell death (Veenman et al., 2010; Kinnally et al., 2011; Veenman et al., 2018; Zeineh et al., 2019; Dimitrova-Shumkovska et al., 2020). Besides our predictions of TSPO functions, a recent study by Yasin et al. reported that TSPO regulates nuclear gene expression mainly through the mitochondria-to-nucleus signalling pathway (Yasin et al., 2017). Our analysis reporting the roles of TSPO in various disease states contributes to the existing knowledge of the effect of TSPO on gene expression.

## Conclusion

This study identifies several canonical signalling pathways and proteins that interact with TSPOAP1 and TSPO, and also reveals the differences in the roles and functions of TSPOAP1 and TSPO. In addition, our data projects the potential roles of TSPOAP1 and TSPO in various diseases. These results provide guidance for experimental analyses of the mechanisms of action and functions of TSPOAP1, which are relatively unclear, particularly in comparison with its counterpart TSPO. Further research on TSPOAP1 could pave a way for the design and development of ligands targeting TSPOAP1 for the treatment of inflammatory diseases and cancer. At the same time, the development of TSPOAP1 selective ligands and biological assay for screening these ligands will help in revealing the pharmacological and biological actions of TSPOAP1. Based on our findings, IPA offers identification of several novel leads to study the detailed mechanisms and functioning of TSPOAP1 in human diseases, however, since these analytical predictions are derived from a myriad of context dependent experimental results stored in the Ingenuity Knowledge Base, the IPA outcomes may not always be emulated in a specific experimental set-up.

## Data Availability

The datasets presented in this study can be found in online repositories. The names of the repository/repositories and accession number(s) can be found in the article/Supplementary Material.
