# Supplementary material for: Bioinformatic Analyses of Canonical Pathways of TSPOAP1 and its Roles in Human Diseases
Source: Front Mol Biosci. 2021 Jun 15;8:667947. doi: 10.3389/fmolb.2021.667947 (PMC8239723; doi:10.3389/fmolb.2021.667947)
Supplement: Supplementary file 1 [file DataSheet1.PDF]

# Supplementary Data

## Bioinformatic Analyses of Canonical Pathways of TSPOAP1 and its Roles in Human Diseases

Sharad Kumar Suthar[1,2]†, Mohammand Maqusood Alam[3]†, Jihye Lee[1], Jitender Monga[4], Alex Joseph[2], Sang-Yoon Lee[1,5]*

[1]Neuroscience Research Institute, Gachon University, Incheon 20565, South Korea

[2]Manipal College of Pharmaceutical Sciences, Manipal University, Manipal 576104, India

[3]Medicinal Chemistry, Institut Pasteur Korea, 16, Daewangpangyo-ro 712 beon-gil, Bundang-gu, Seongnam-si, Gyeonggi-do 13488, South Korea

[4]Department of Urology, Postgraduate Institute of Medical Education and Research, Chandigarh 160012, India.

[5]Department of Neuroscience, College of Medicine, Gachon University, Incheon 21936, South Korea

*Corresponding author: Prof. Dr. Sang-Yoon Lee, Tel.: +82-10-7317-5391, E-mail: rchemist@gachon.ac.kr
(†Authors contributed equally)

## Contents

**Table S1.** The dataset of genes associated with TSPO associated protein 1 (TSPOAP1) used for Ingenuity Pathway Analysis
**Table S2.** The dataset of genes associated with TSPO used for Ingenuity Pathway Analysis
**Table S3.** Human genes common between TSPOAP1 and TSPO
**Table S4.** Canonical pathways and interaction networks of TSPO predicted by Ingenuity Pathway Analysis
**Figure S1.** Canonical pathways of TSPOAP1 and TSPO predicted by Ingenuity Pathway Analysis
**Figure S2.** Overlapping of top-ten canonical pathways of TSPOAP1
**Figure S3.** Overlapping of top-ten canonical pathways of TSPO
**Figure S4.** Necroptosis signalling pathway of TSPOAP1 and TSPO
**Figure S5.** Sirtulin signalling pathway of TSPOAP1 and TSPO in the cytoplasm
**Figure S6.** Sirtulin signalling pathway of TSPOAP1 and TSPO in the nucleus
**Figure S7.** Mitochondrial dysfunction pathway of TSPOAP1 and TSPO
**Figure S8.** Inflammasome pathway of TSPOAP1 and TSPO
**Figure S9.** The heat map diagram showing the diseases and functions of TSPOAP1
**Figure S10.** Top-ranked diseases and disorders of TSPOAP1 predicted by Ingenuity Pathway Analysis
**Figure S11.** The heat map diagram showing the diseases and functions of TSPO
**Figure S12.** Top-ranked diseases and disorders of TSPO predicted by Ingenuity Pathway Analysis

**Table S1.** The dataset of genes associated with TSPO associated protein 1 (TSPOAP1) used for Ingenuity Pathway Analysis.

| **TSPO associated protein 1 gene data set** | | | | |
|---|---|---|---|---|
| **No.** | **Organism** | **Gene ID** | **Name** | **Aliases** |
| 1 | *Homo sapiens* | 706 | TSPO | BPBS, BZRP, DBI, IBP, MBR, PBR, PBS, PKBS, PTBR, mDRC, pk18 |
| 2 | *Homo sapiens* | 114548 | NLRP3 | AGTAVPRL, AII, AVP, C1orf7, CIAS1, CLR1.1, DFNA34, FCAS, FCAS1, FCU, KEFH, MWS, NALP3, PYPAF1 |
| 3 | *Homo sapiens* | 4982 | TNFRSF11B | OCIF, OPG, PDB5, TR1 |
| 4 | *Homo sapiens* | 2876 | GPX1 | GPXD, GSHPX1 |
| 5 | *Homo sapiens* | 7412 | VCAM1 | CD106, INCAM-100 |
| 6 | *Homo sapiens* | 5591 | PRKDC | DNA-PKC, DNA-PKcs, DNAPK, DNAPKc, DNPK1, HYRC, HYRC1, IMD26, XRCC7, p350 |
| 7 | *Homo sapiens* | 9256 | TSPOAP1 | BZRAP1, PBR-IP, PRAX-1, PRAX1, RIM-BP1, RIMBP1 |
| 8 | *Homo sapiens* | 7416 | VDAC1 | PORIN, VDAC-1 |
| 9 | *Homo sapiens* | 24145 | PANX1 | MRS1, OOMD7, PX1, UNQ2529 |
| 10 | *Homo sapiens* | 7083 | TK1 | TK2 |
| 11 | *Homo sapiens* | 498 | ATP5F1A | ATP5A, ATP5A1, ATP5AL2, ATPM, COXPD22, HEL-S-123m, MC5DN4, MOM2, OMR, ORM, hATP1 |
| 12 | *Homo sapiens* | 10554 | AGPAT1 | 1-AGPAT1, G15, LPAAT-alpha, LPAATA |
| 13 | *Homo sapiens* | 1622 | DBI | ACBD1, ACBP, CCK-RP, EP |
| 14 | *Homo sapiens* | 9479 | MAPK8IP1 | IB1, JIP-1, JIP1, PRKM8IP |
| 15 | *Homo sapiens* | 285 | ANGPT2 | AGPT2, ANG2 |
| 16 | *Homo sapiens* | 55847 | CISD1 | C10orf70, MDS029, ZCD1, mitoNEET |
| 17 | *Homo sapiens* | 1381 | CRABP1 | CRABP, CRABP-I, CRABPI, RBP5 |
| 18 | *Homo sapiens* | 90993 | CREB3L1 | OASIS, OI16 |
| 19 | *Homo sapiens* | 374383 | NCR3LG1 | B7-H6, B7H6, DKFZp686O24166 |
| 20 | *Homo sapiens* | 5020 | OXT | OT, OT-NPI-NPI, OXT |
| 21 | *Homo sapiens* | 57463 | AMIGO1 | ALI2, AMIGO, AMIGO-1 |
| 22 | *Homo sapiens* | 284348 | LYPD5 | PRO4356 |
| 23 | *Homo sapiens* | 5817 | PVR | CD155, HVED, NECL5, Necl-5, PVS, TAGE4 |
| 24 | *Homo sapiens* | 7182 | NR2C2 | TAK1, TR4 |
| 25 | *Homo sapiens* | 2207 | FCER1G | FCRG |
| 26 | *Homo sapiens* | 3385 | ICAM3 | CD50, CDW50, ICAM-R |
| 27 | *Homo sapiens* | 7417 | VDAC2 | POR |
| 28 | *Homo sapiens* | 6047 | RNF4 | RES4-26, SLX5, SNURF |
| 29 | *Homo sapiens* | 160728 | SLC5A8 | AIT, SMCT, SMCT1 |
| 30 | *Homo sapiens* | 515 | ATP5PB | ATP5F1, PIG47 |
| 31 | *Homo sapiens* | 7419 | VDAC3 | HD-VDAC3, VDAC-3 |
| 32 | *Homo sapiens* | 378 | ARF4 | ARF2 |
| 33 | *Homo sapiens* | 4223 | MEOX2 | GAX, MOX2 |
| 34 | *Homo sapiens* | 539 | ATP5PO | ATP5O, ATPO, HMC08D05, OSCP |
| 35 | *Homo sapiens* | 222662 | LHFPL5 | DFNB67, TMHS, dJ510O8.8 |

*Taxonomy ID: 9606 for all genes*

**Table S2.** The dataset of genes associated with TSPO used for Ingenuity Pathway Analysis.

| TSPO gene data set | | | | |
|---|---|---|---|---|
| **No.** | **Organism** | **Gene ID** | **Name** | **Aliases** |
| 1 | *Homo sapiens* | 706 | TSPO | BPBS, BZRP, DBI, IBP, MBR, PBR, PBS, PKBS, PTBR, mDRC, pk18 |
| 2 | *Homo sapiens* | 9256 | TSPOAP1 | BZRAP1, PBR-IP, PRAX-1, PRAX1, RIM-BP1, RIMBP1 |
| 3 | *Homo sapiens* | 4982 | TNFRSF11B | OCIF, OPG, PDB5, TR1 |
| 4 | *Homo sapiens* | 114548 | NLRP3 | AGTAVPRL, AII, AVP, C1orf7, CIAS1, CLR1.1, DFNA34, FCAS, FCAS1, FCU, KEFH, MWS, NALP3, PYPAF1 |
| 5 | *Homo sapiens* | 7412 | VCAM1 | CD106, INCAM-100 |
| 6 | *Homo sapiens* | 5591 | PRKDC | DNA-PKC, DNA-PKcs, DNAPK, DNAPKc, DNPK1, HYRC, HYRC1, IMD26, XRCC7, p350 |
| 7 | *Homo sapiens* | 285 | ANGPT2 | AGPT2, ANG2 |
| 8 | *Homo sapiens* | 2876 | GPX1 | GPXD, GSHPX1 |
| 9 | *Homo sapiens* | 7416 | VDAC1 | PORIN, VDAC-1 |
| 10 | *Homo sapiens* | 5020 | OXT | OT, OT-NPI-NPI, OXT |
| 11 | *Homo sapiens* | 7083 | TK1 | TK2 |
| 12 | *Homo sapiens* | 5817 | PVR | CD155, HVED, NECL5, Necl-5, PVS, TAGE4 |
| 13 | *Homo sapiens* | 7182 | NR2C2 | TAK1, TR4 |
| 14 | *Homo sapiens* | 24145 | PANX1 | MRS1, OOMD7, PX1, UNQ2529 |
| 15 | *Homo sapiens* | 498 | ATP5F1A | ATP5A, ATP5A1, ATP5AL2, ATPM, COXPD22, HEL-S-123m, MC5DN4, MOM2, OMR, ORM, hATP1 |
| 16 | *Homo sapiens* | 3385 | ICAM3 | CD50, CDW50, ICAM-R |
| 17 | *Homo sapiens* | 7417 | VDAC2 | POR |
| 18 | *Homo sapiens* | 1622 | DBI | ACBD1, ACBP, CCK-RP, EP |
| 19 | *Homo sapiens* | 6047 | RNF4 | RES4-26, SLX5, SNURF |
| 20 | *Homo sapiens* | 9479 | MAPK8IP1 | IB1, JIP-1, JIP1, PRKM8IP |
| 21 | *Homo sapiens* | 160728 | SLC5A8 | AIT, SMCT, SMCT1 |
| 22 | *Homo sapiens* | 55847 | CISD1 | C10orf70, MDS029, ZCD1, mitoNEET |
| 23 | *Homo sapiens* | 2207 | FCER1G | FCRG |
| 24 | *Homo sapiens* | 7419 | VDAC3 | HD-VDAC3, VDAC-3 |
| 25 | *Homo sapiens* | 378 | ARF4 | ARF2 |
| 26 | *Homo sapiens* | 90993 | CREB3L1 | OASIS, OI16 |
| 27 | *Homo sapiens* | 1381 | CRABP1 | CRABP, CRABP-I, CRABPI, RBP5 |
| 28 | *Homo sapiens* | 4223 | MEOX2 | GAX, MOX2 |
| 29 | *Homo sapiens* | 374383 | NCR3LG1 | B7-H6, B7H6, DKFZp686O24166 |
| 30 | *Homo sapiens* | 539 | ATP5PO | ATP5O, ATPO, HMC08D05, OSCP |
| 31 | *Homo sapiens* | 4294 | MAP3K10 | MEKK10, MLK2, MST |
| 32 | *Homo sapiens* | 515 | ATP5PB | ATP5F1, PIG47 |
| 33 | *Homo sapiens* | 222642 | TSPO2 | BZRPL1 |
| 34 | *Homo sapiens* | 10462 | CLEC10A | CD301, CLECSF13, CLECSF14, HML, HML2, MGL |
| 35 | *Homo sapiens* | 154091 | SLC2A12 | GLUT12, GLUT8 |
| 36 | *Homo sapiens* | 10554 | AGPAT1 | 1-AGPAT1, G15, LPAAT-alpha, LPAATA |
| 37 | *Homo sapiens* | 10632 | ATP5MG | ATP5JG, ATP5L |
| 38 | *Homo sapiens* | 222662 | LHFPL5 | DFNB67, TMHS, dJ510O8.8 |
| 39 | *Homo sapiens* | 252839 | TMEM9 | DERM4A, TMEM9 |
| 40 | *Homo sapiens* | 284348 | LYPD5 | PRO4356 |
| 41 | *Homo sapiens* | 57463 | AMIGO1 | ALI2, AMIGO, AMIGO-1 |
| 42 | *Homo sapiens* | 342900 | LEUTX | - |
| 43 | *Homo sapiens* | 3754 | KCNF1 | IK8, KCNF, KV5.1, kH1 |
| 44 | *Homo sapiens* | 81853 | TMEM14B | - |
| 45 | *Homo sapiens* | 200232 | FAM209A | C20orf106, dJ1153D9.3 |
| 46 | *Homo sapiens* | 6775083 | COX1 | - |

*Taxonomy ID: 9606 for all genes*

**Table S3.** Human genes common between TSPOAP1 and TSPO.

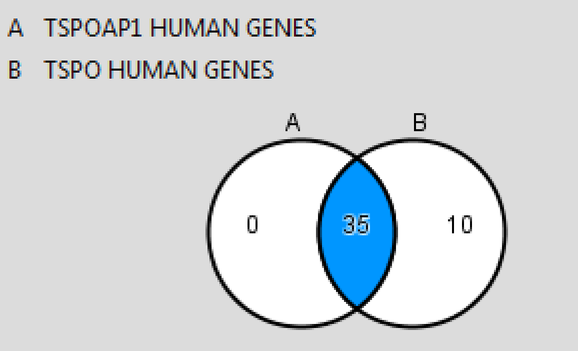


| No. | Genes common between TSPOAP1 and TSPO | Other genes related to TSPO |
|---|---|---|
| 1 | AGPAT1 | ATP5MG |
| 2 | ARF4 | CLEC10A |
| 3 | ATP5F1A | FAM209A |
| 4 | ATP5PO | KCNF1 |
| 5 | CISD1 | LEUTX |
| 6 | CRABP1 | MAP3K10 |
| 7 | CREB1 | SLC2A12 |
| 8 | CREB3L1 | TMEM14B |
| 9 | DBI | TMEM9 |
| 10 | FCER1G | TSPO2 |
| 11 | GPX1 | |
| 12 | ICAM3 | |
| 13 | LHFPL5 | |
| 14 | LYPD5 | |
| 15 | MAPK8IP1 | |
| 16 | MEOX2 | |
| 17 | NCR3LG1 | |
| 18 | NLRP3 | |
| 19 | NR2C2 | |
| 20 | OXT | |
| 21 | PANX1 | |
| 22 | PRKDC | |
| 23 | PVR | |
| 24 | RNF4 | |
| 25 | SELL | |
| 26 | SLC5A8 | |
| 27 | TK1 | |
| 28 | TNFRSF11B | |
| 29 | TSPO | |
| 30 | TSPOAP1 | |
| 31 | Uox | |
| 32 | VCAM1 | |
| 33 | VDAC1 | |
| 34 | VDAC2 | |
| 35 | VDAC3 | |

**Table S4.** Canonical pathways and interaction networks of TSPO predicted by Ingenuity Pathway Analysis.

| **Rank** | **Canonical pathway** | **-log *P*-value** | **Interacting pathway proteins/genes** |
|---|---|---|---|
| 1 | Necroptosis signalling pathway | 6,09 | NLRP3, TNFRSF11B, TSPO, VDAC1,VDAC2,VDAC3 |
| 2 | Mitochondrial Dysfunction | 5,9 | Mitochondrial complex V or Mitochondrial ATP synthase (ATP5F1A, ATP5MG, ATP5PO)<br>VDAC1, VDAC2, VDAC3 |
| 3 | Sirtuin Signaling Pathway | 4,56 | ATP5F1A, PRKDC (DNA-PK), TSPO, VDAC1,VDAC2,VDAC3 |
| 4 | Inflammasome pathway | 3,09 | NLRP3,PANX1 |
| 5 | SAPK/JNK Signaling | 2,89 | FCER1G, MAP3K10, MAPK8IP1 |
| 6 | Oxidative Phosphorylation | 2,86 | Mitochondrial complex V or Mitochondrial ATP synthase (ATP5F1A, ATP5MG, ATP5PO) |
| 7 | Granulocyte Adhesion and Diapedesis | 2,29 | SELL (L-Selectin), TNFRSF11B, VCAM1 |
| 8 | Dendritic Cell Maturation | 2,23 | CREB1, FCER1G, TNFRSF11B |
| 9 | Natural Killer Cell Signaling | 2,12 | FCER1G, MAP3K10, PVR |
| 10 | T Helper Cell Differentiation | 2,03 | FCER1G, TNFRSF11B |
| 11 | Huntington's Disease Signaling | 1,88 | ATP5F1A, CREB1, MAP3K10 |
| 12 | Salvage Pathways of Pyrimidine Deoxyribonucleotides | 1,77 | TK1 |
| 13 | Type I Diabetes Mellitus Signaling | 1,67 | FCER1G, TNFRSF11B |
| 14 | Neuroinflammation Signaling Pathway | 1,63 | CREB1, NLRP3, VCAM1 |
| 15 | NGF Signaling | 1,62 | CREB1, MAP3K10 |
| 16 | Role of Macrophages, Fibroblasts and Endothelial Cells in Rheumatoid Arthritis | 1,59 | CREB1, TNFRSF11B, VCAM1 |
| 17 | DNA Double-Strand Break Repair by Non-Homologous End Joining | 1,53 | PRKDC |
| 18 | Reelin Signaling in Neurons | 1,52 | MAP3K10, MAPK8IP1 |
| 19 | White Adipose Tissue Browning Pathway | 1,52 | CREB1, FCER1G |
| 20 | Estrogen Receptor Signaling | 1,52 | ATP5F1A, CREB1, PRKDC (DNA-PK) |
| 21 | Granzyme B Signaling | 1,48 | PRKDC (DNA-PK) |
| 22 | Role of Pattern Recognition Receptors in Recognition of Bacteria and Viruses | 1,43 | CREB1, NLRP3 |
| 23 | PKCθ Signaling in T Lymphocytes | 1,4 | FCER1G, MAP3K10 |
| 24 | Hepatic Fibrosis Signaling Pathway | 1,39 | CREB1, TNFRSF11B, VCAM1 |
| 25 | CDP-diacylglycerol Biosynthesis I | 1,38 | AGPAT1 |
| 26 | HMGB1 Signaling | 1,36 | TNFRSF11B, VCAM1 |
| 27 | Glutathione Redox Reactions I | 1,34 | GPX1 |
| 28 | Phosphatidylglycerol Biosynthesis II (Non-plastidic) | 1,34 | AGPAT1 |
| 29 | Lipid Antigen Presentation by CD1 | 1,3 | FCER1G |

**NALP3**; Nucleotide-binding oligomerization domain, leucine rich repeat and pyrin domain containing 3,**TNFR1**; Tumor necrosis factor receptor 1, **VDAC1/2/3**; Voltage-dependent anion-selective channel 1/2/3**, ATP5F1A**; ATP synthase F1 subunit alpha,**DNA-PK (PRKDC)**; DNA-dependent protein kinase,**PANX1**; Pannexin 1, **MAP3K10**; Mitogen-activated protein kinase kinase kinase 10, **MAPK8IP1**; Mitogen-Activated Protein Kinase 8 Interacting Protein 1, **SELL**; L-Selectin, **CREB1**; Cyclic AMP response element binding protein 1, **PVR**; PVR cell adhesion molecule**, TK1**; Thymidine kinase 1, **VCAM-1**; Vascular cell adhesion molecule 1, **AGPAT1**; 1-Acylglycerol-3-phosphate-O-acyltransferase, **GPX1**; Glutathione peroxidase 1.

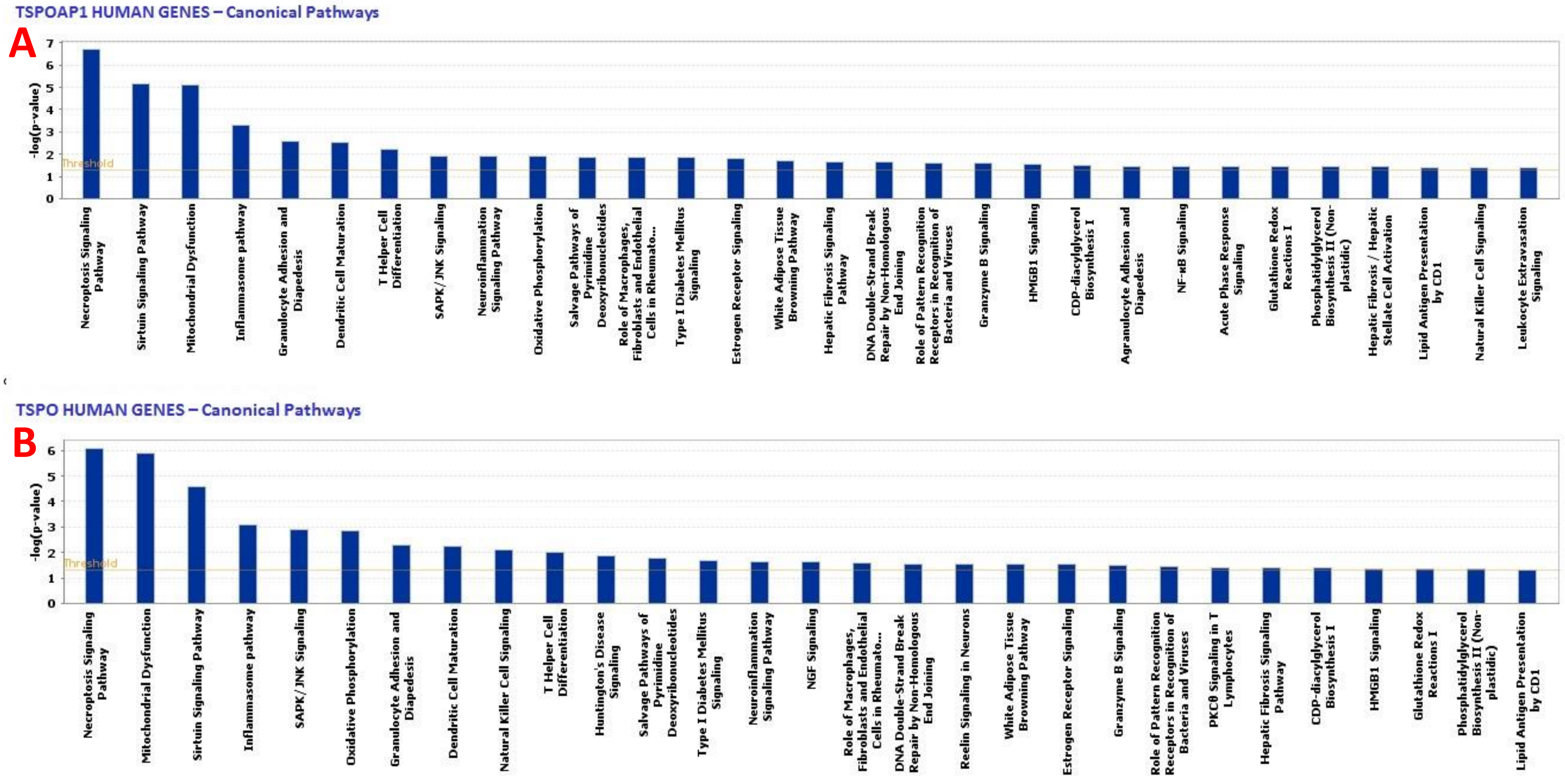


**Figure S1.** Canonical pathways of TSPOAP1 and TSPO predicted by Ingenuity Pathway Analysis. Pathways were ranked based on their *P* values.

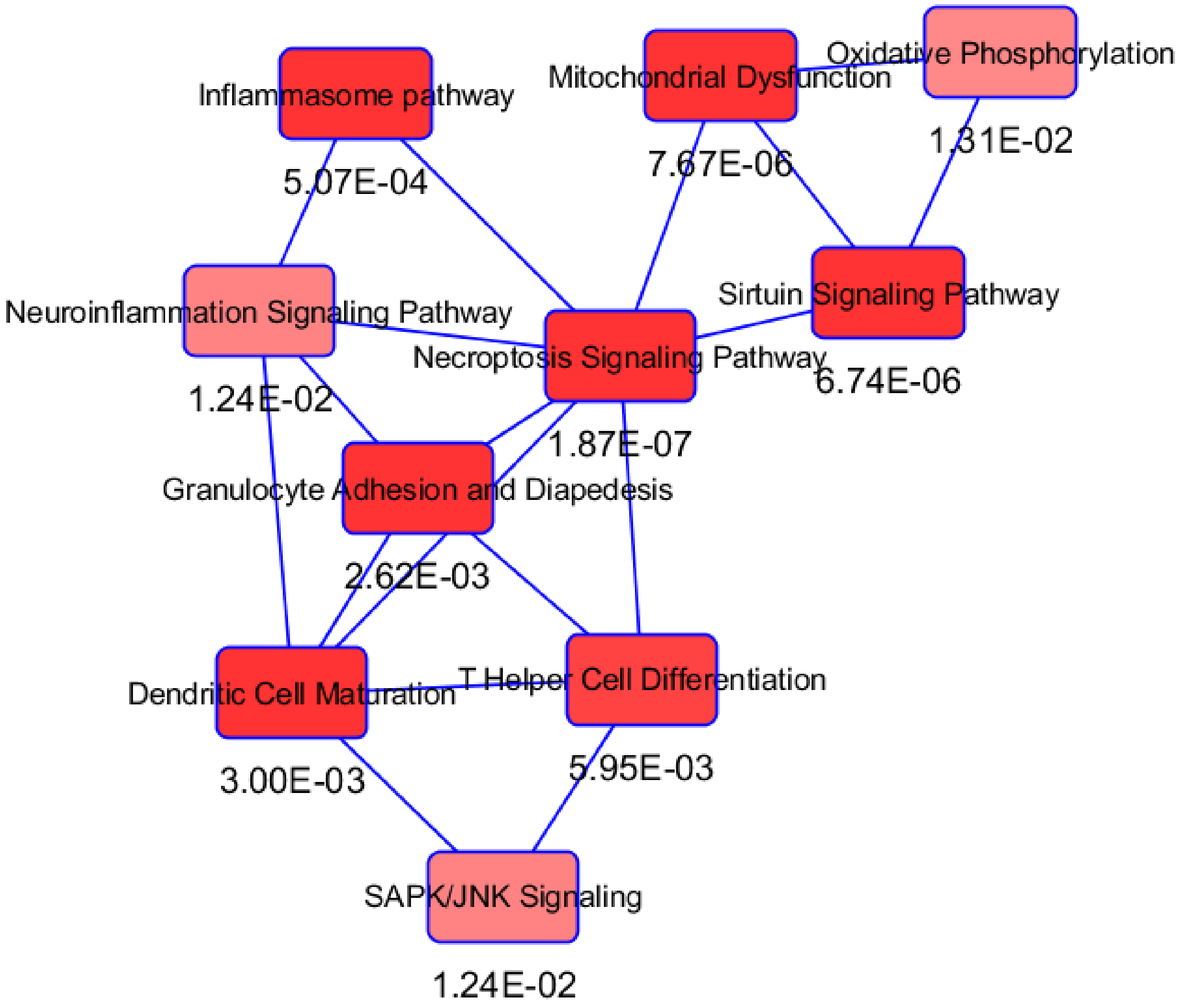


**Figure S2.** Overlapping of top-ten canonical pathways of TSPOAP1. A low *P*-value indicates higher overlapping.

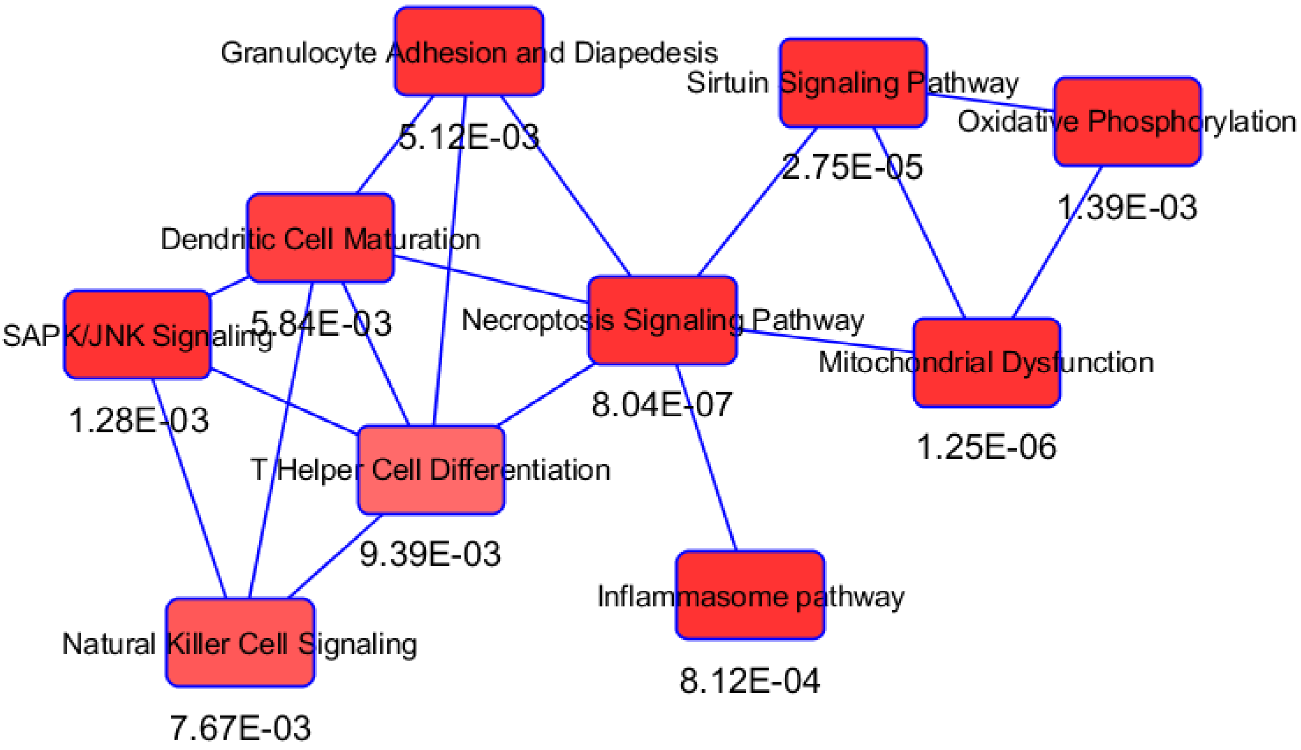


**Figure S3.** Overlapping of top-ten canonical pathways of TSPO. A low *P*-value indicates higher overlapping.

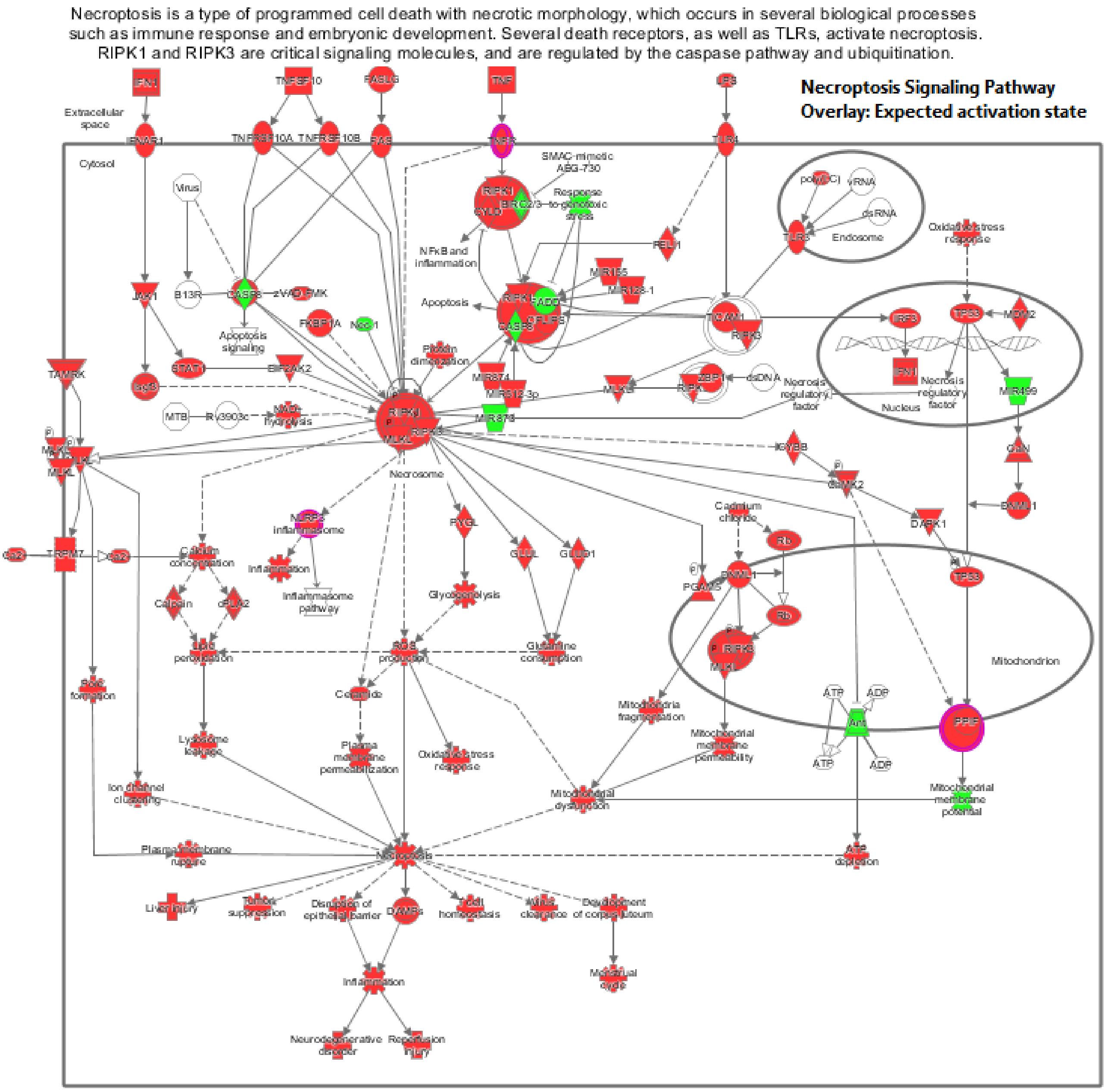


**Figure S4.** Necroptosis signalling pathway of TSPOAP1 and TSPO. Red color indicates up-regulation, whereas green color indicates down-regulation of proteins or genes.

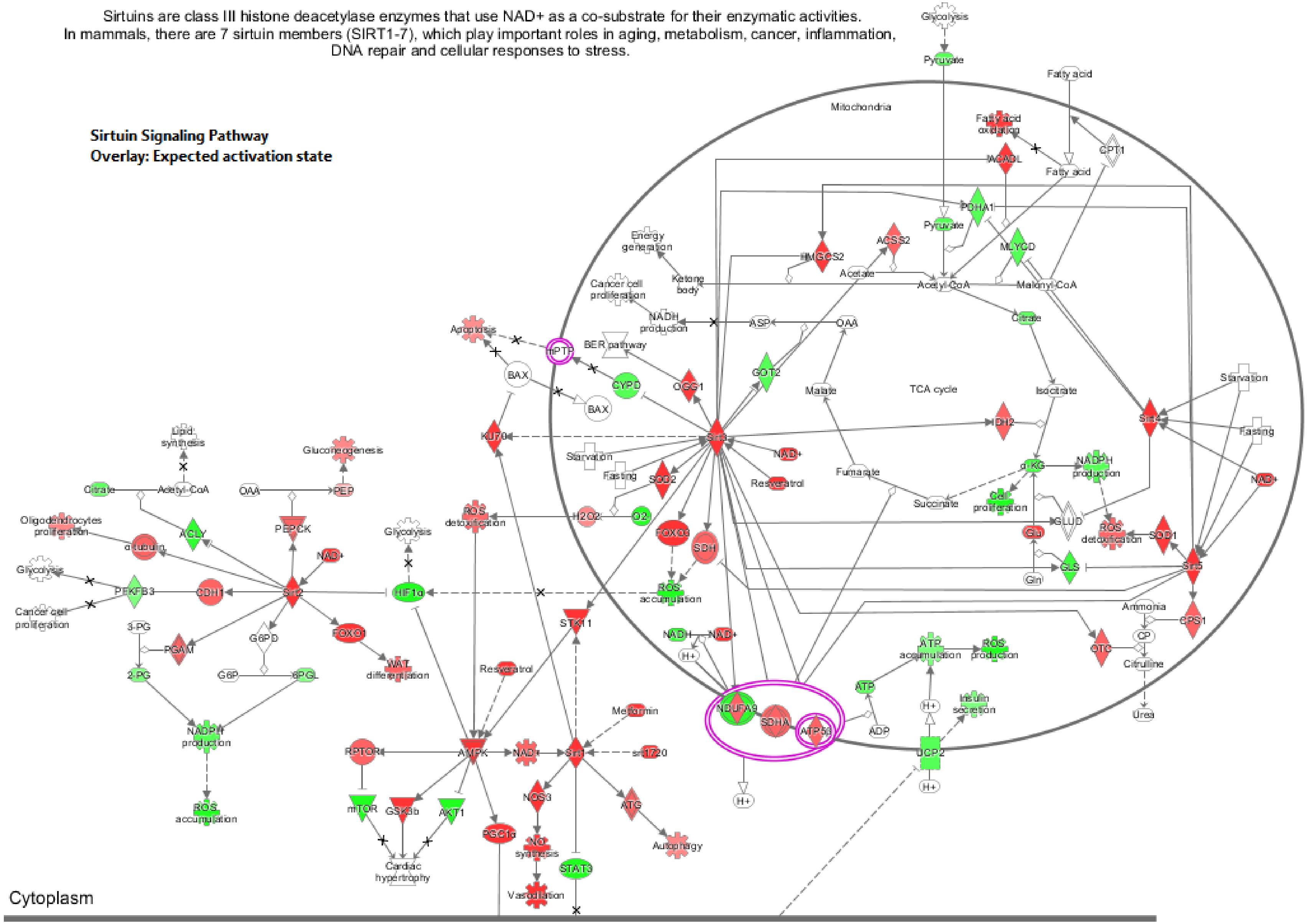


**Figure S5.** Sirtulin signalling pathway of TSPOAP1 and TSPO in the cytoplasm. Red color indicates up-regulation, whereas green color indicates down-regulation of proteins or genes.

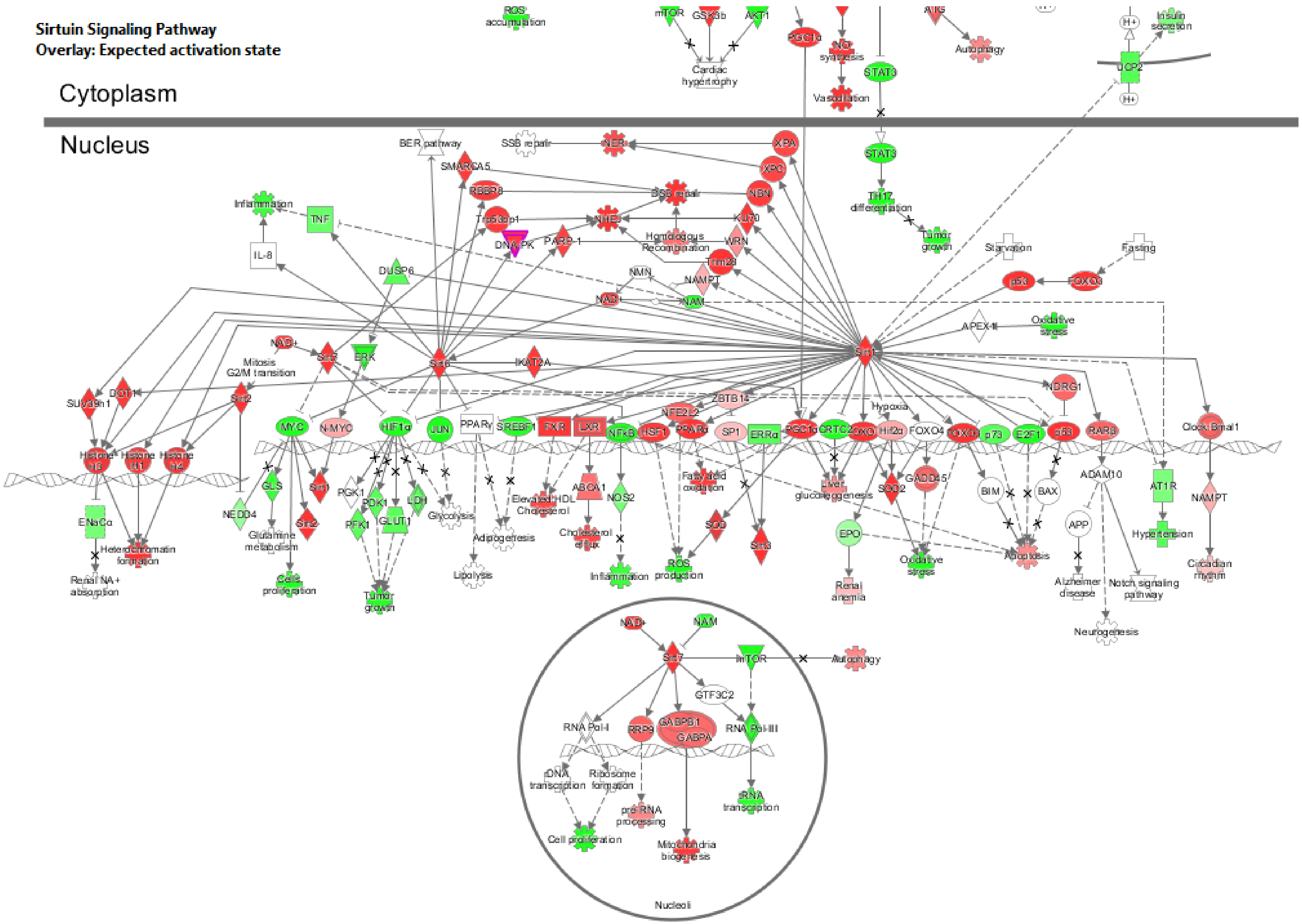


**Figure S6.** Sirtulin signalling pathway of TSPOAP1 and TSPO in the nucleus. Red color indicates up-regulation, whereas green color indicates down-regulation of proteins or genes.

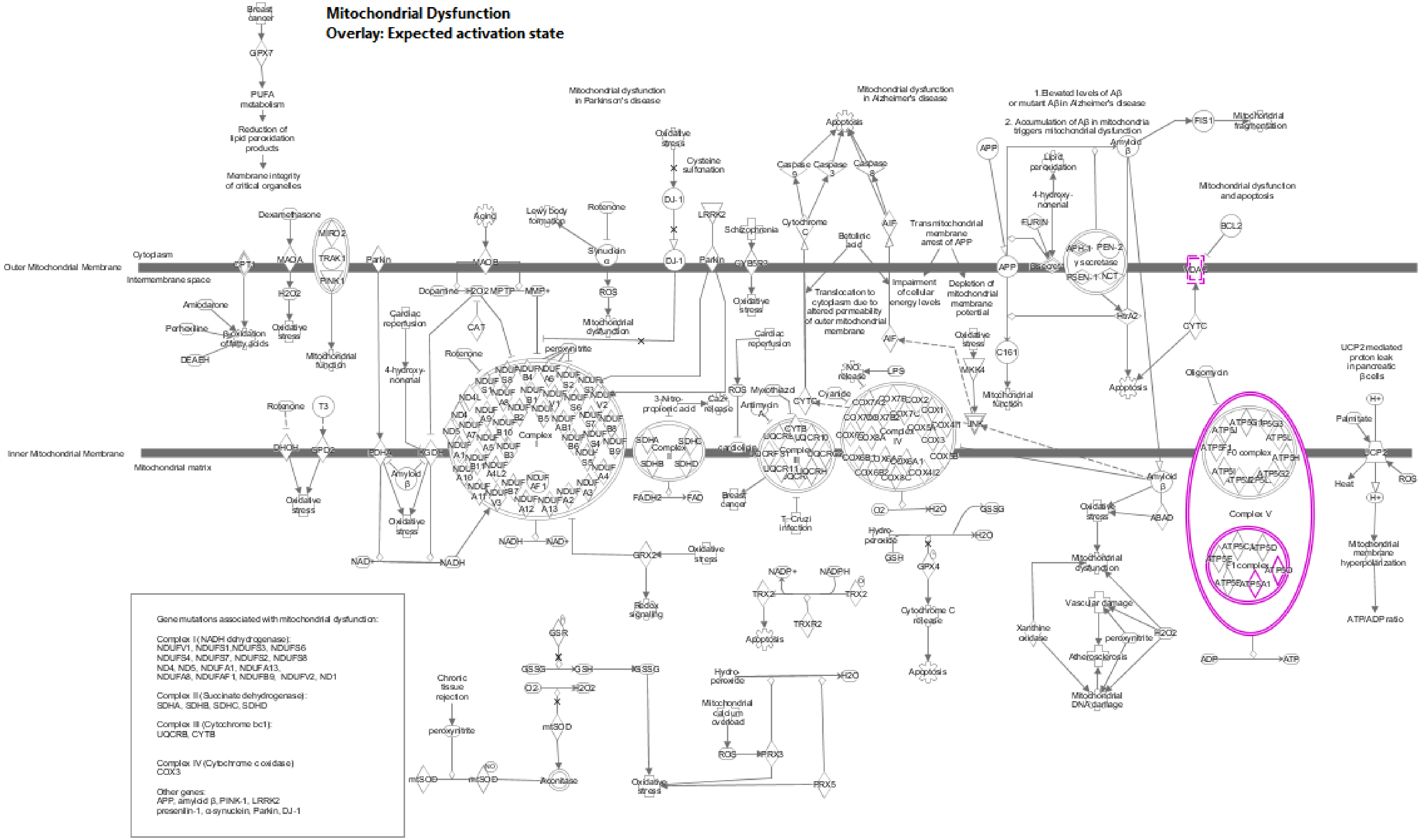


**Figure S7.** Mitochondrial dysfunction pathway of TSPOAP1 and TSPO.

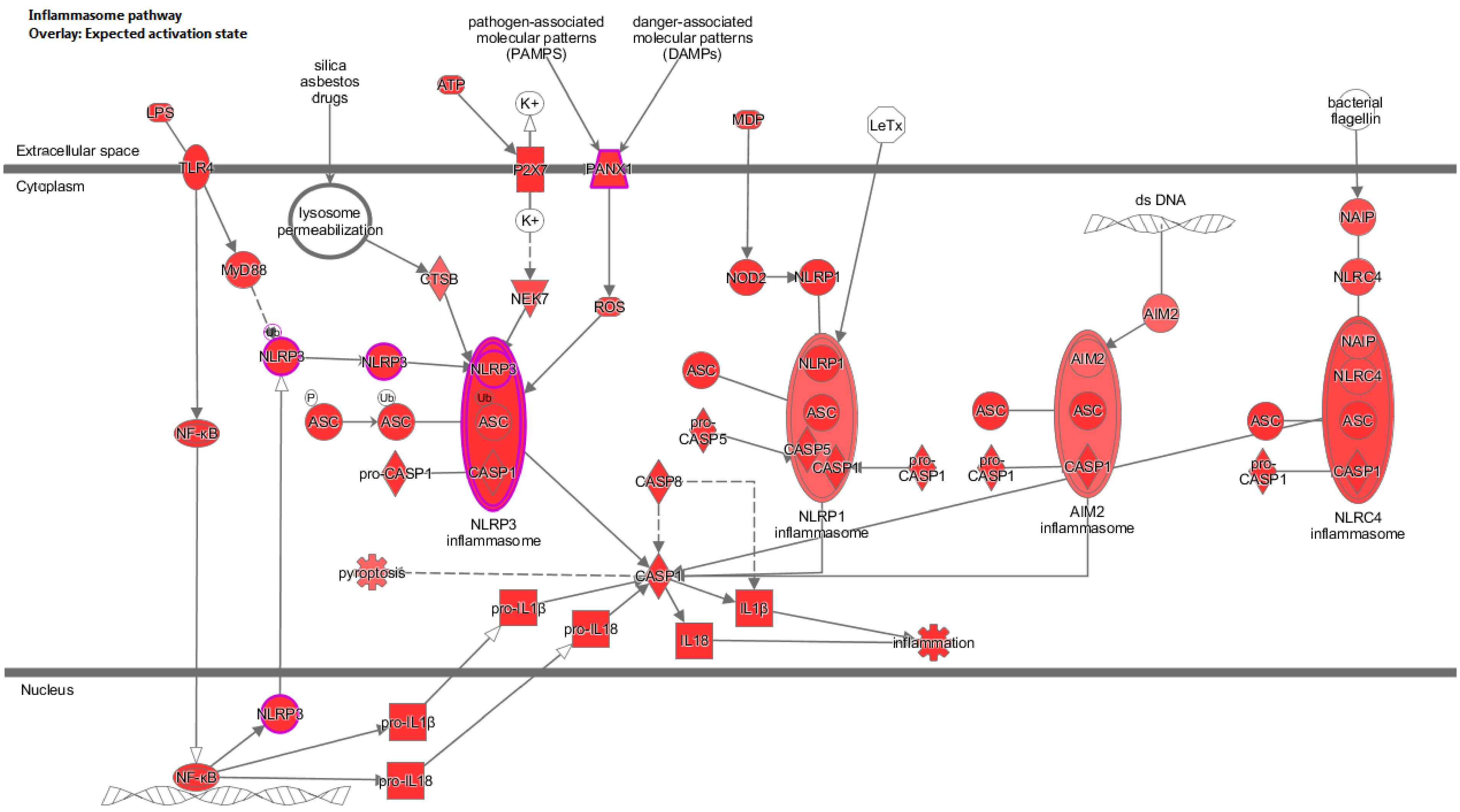


**Figure S8.** Inflammasome pathway of TSPOAP1 and TSPO. Red color indicates up-regulation of proteins or genes.

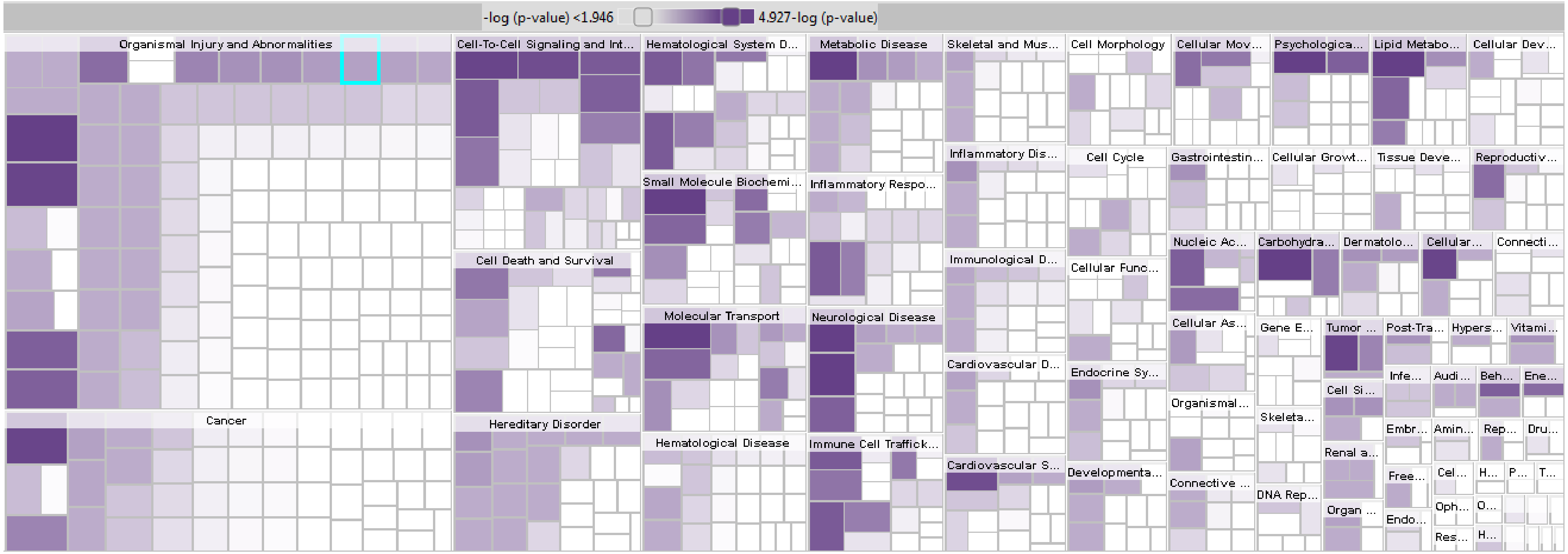


**Figure S9.** The heat map diagram showing the diseases and functions of TSPOAP1. The low *P*-value (deep color) indicates less likelihood of results by chance.

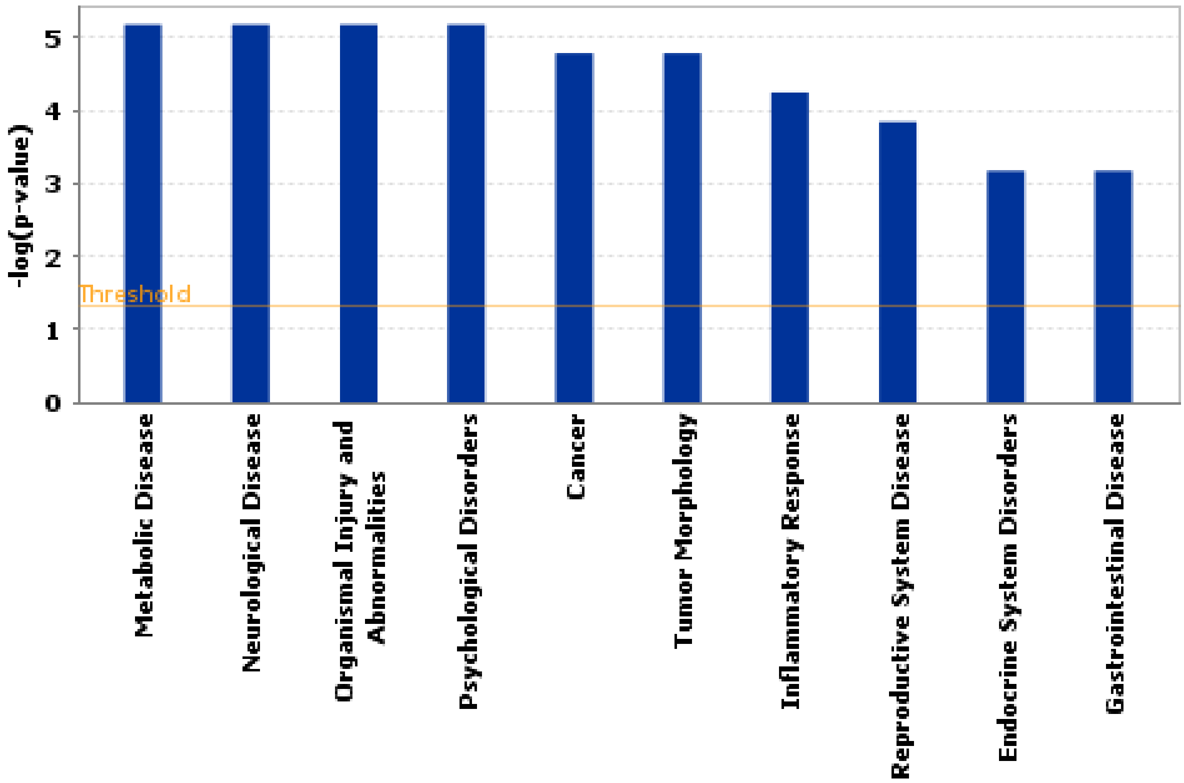


**Figure S10.** Top-ranked diseases and disorders of TSPOAP1 predicted by Ingenuity Pathway Analysis. The results are ranked based on their *P* values.

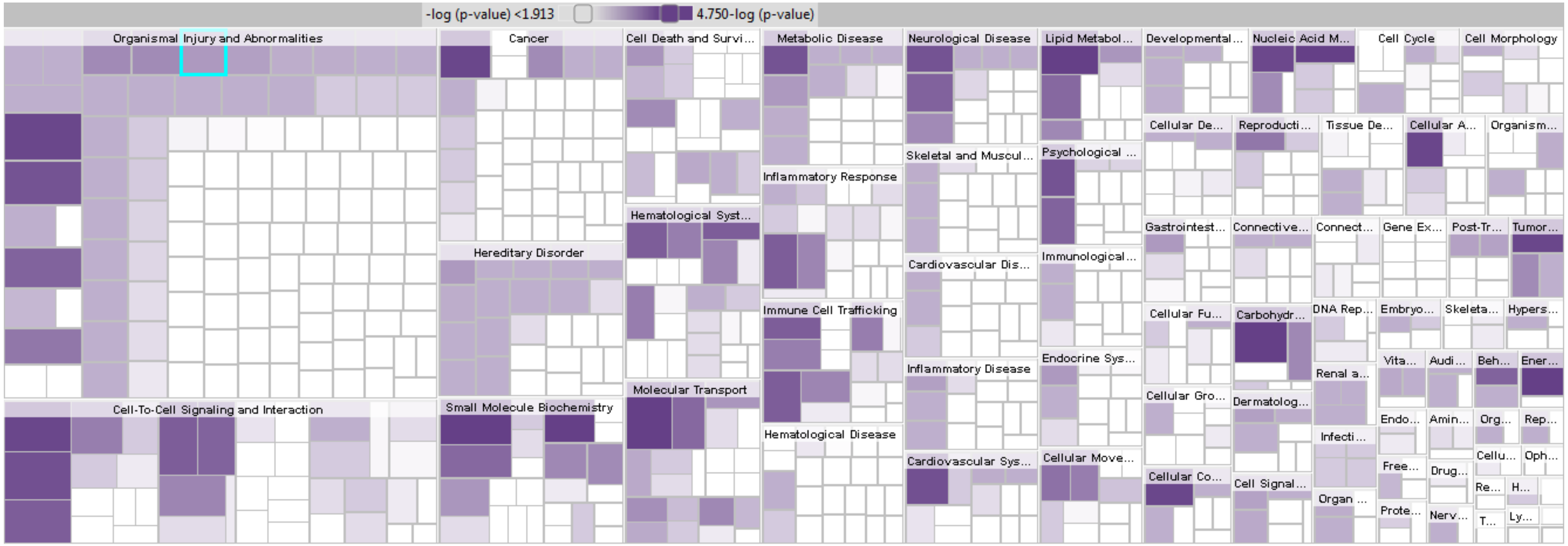


**Figure S11.** The heat map diagram showing the diseases and functions of TSPO. The low *P*-value (deep color) indicates less likelihood of results by chance.

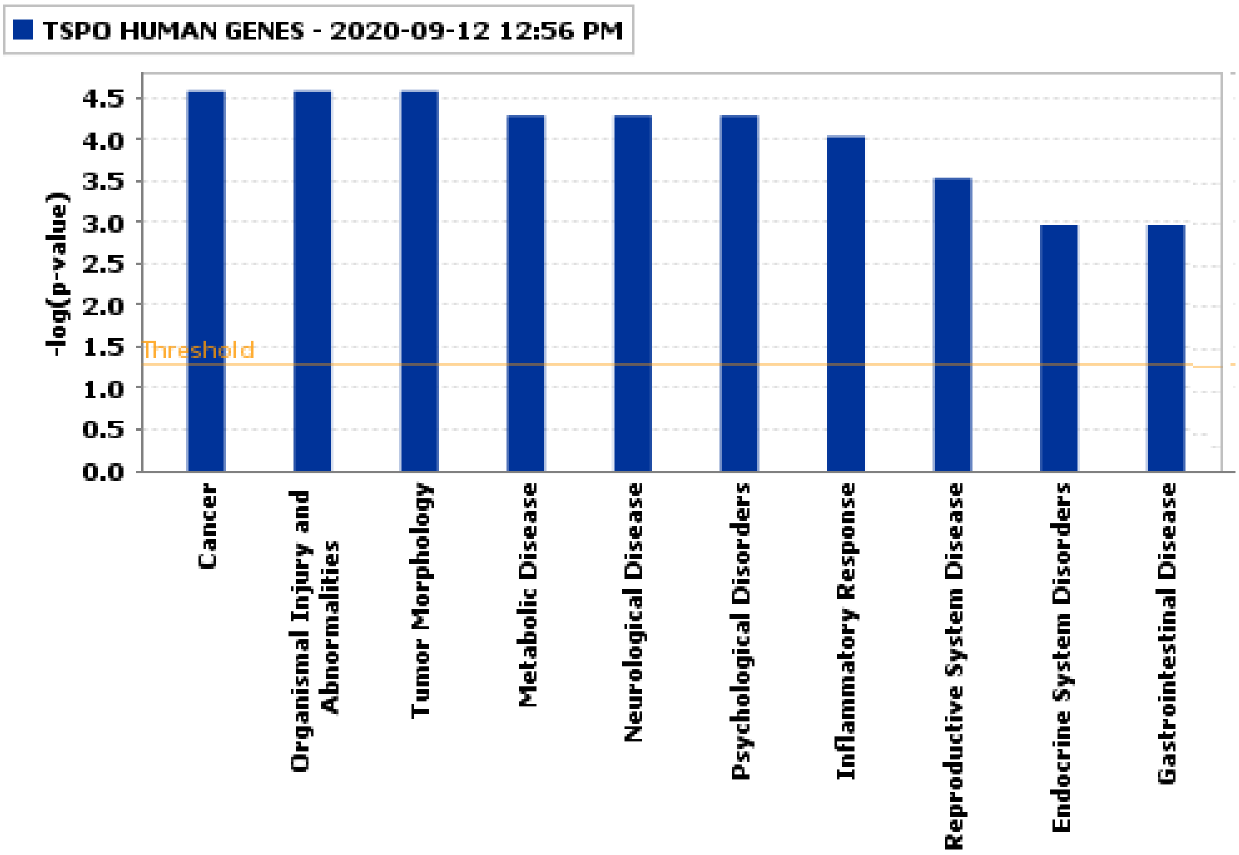


**Figure S12.** Top-ranked diseases and disorders of TSPO predicted by Ingenuity Pathway Analysis. The results are ranked based on their *P* values.
